# Dual Endoscopic Approach for Radiation-Induced Complete Esophageal Obstruction in the Upper Esophagus: Insights From 2 Challenging Cases

**DOI:** 10.14309/crj.0000000000002087

**Published:** 2026-05-18

**Authors:** Mehreen Siyal, Arif R. Siddiqui, Shanil Kadir, Saad Khalid Niaz

**Affiliations:** 1 Sindh Institute of Advanced Endoscopy and Gastroenterology (SIAG), Civil Hospital, Karachi, Pakistan

**Keywords:** complete esophageal obstruction, radiation-induced strictures, combined antegrade and retrograde endoscopic dilatation, transillumination, endoscopic recanalization, oral intake recovery, quality of life

## Abstract

Complete esophageal obstruction (CEO) is a rare complication of chemoradiotherapy for head and neck cancers. We report 2 cases of radiation-induced CEO in the upper esophagus, managed with combined antegrade and retrograde endoscopic dilatation (CARD). In 1 case, percutaneous endoscopic gastrostomy (PEG) dependence was eliminated, and swallowing was improved to a soft diet. In the second case, recurrence required repeat CARD, resulting in partial swallowing recovery but persistent PEG dependence. Both procedures were technically successful without major complications. These cases illustrate that CARD is a safe and effective alternative to surgery in CEO, even in technically challenging sites, enhancing quality of life, although recurrence remains a concern.

## INTRODUCTION

Complete esophageal obstruction (CEO), with an incidence of 0.8%–5%, is a rare but serious complication after radiation therapy for head and neck cancers, leading to severe dysphagia and poor quality of life.^1–3^ Radiotherapy leads to esophageal stricture through several mechanisms, including fibrosis caused by progressive obliterative endarteritis and ischemia of the esophageal wall.^4^ Contributing factors include concurrent chemotherapy, upper esophageal radiation, and high radiation dose.^5^ Dysphagia has detrimental effects on the quality of life in these patients.^6^ Those with CEO are unable to swallow even saliva, necessitating constant spitting and profoundly impairing daily life.^7^ Use of the antegrade approach with wire cannulation and serial dilations can be precarious because of the increased risk of perforation.^8^ Although traditional management involved surgery, combined antegrade and retrograde endoscopic dilatation (CARD) offers a less invasive alternative.^9^ This technique has shown promise for recanalization of strictures, but its application in upper esophageal strictures, particularly those near the upper esophageal sphincter, remains underreported.

We hereby report 2 cases of CEO in the upper esophagus treated with CARD, highlighting technical considerations, outcomes, and limitations. Informed consent was obtained from both patients.

## CASE REPORT

### Case 1

**Figure 1.**
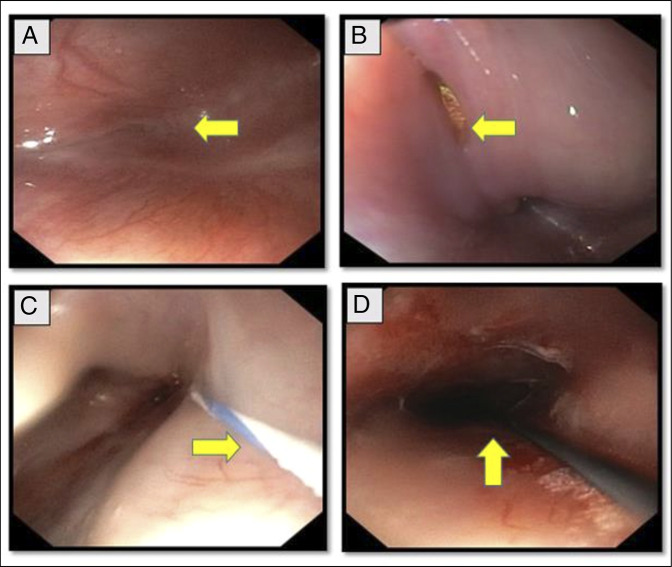
Endoscopic views of antegrade scope in CASE 1 with arrow head showing: (A) complete esophageal obstruction; (B) transillumination of light from retrograde scope; (C) guidewire passed from retrograde scope across the stricture; and (D) recanalization of esophageal lumen after combined antegrade and retrograde endoscopic dilatation procedure.

**Figure 2.**
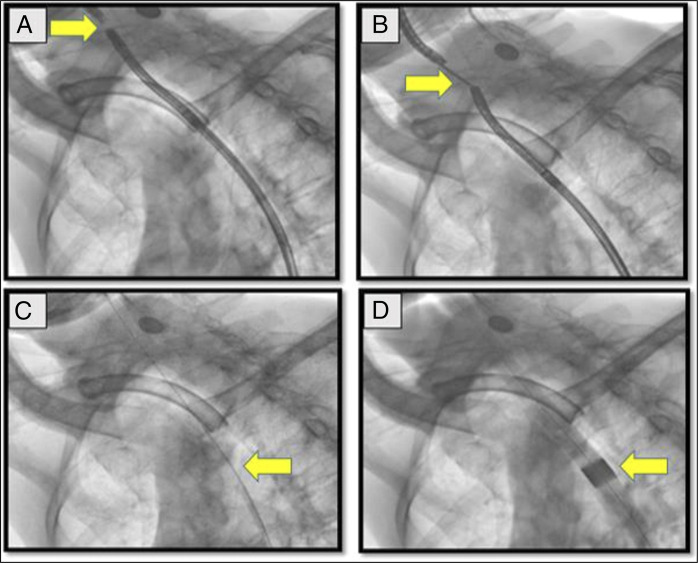
Fluoroscopic images of CASE 1 with arrow head showing: (A) transillumination by approximation of the 2 scopes; (B) two ultrathin gastroscopes in place and grasping forceps from antregrade scope; (C) guidewire in place for dilatation; and (D) dilatation through Savary-Gilliard dilator.

A 54-year-old man with squamous cell carcinoma of the buccal mucosa, previously treated with 5 cycles of chemotherapy and 31 sessions of radiotherapy, presented with a 6-month history of complete dysphagia (Functional Oral Intake Scale [FOIS] score 1). The FOIS, a 7-point scale ranging from 1 (nothing by mouth) to 7 (normal oral intake), was used to assess swallowing function, with a score of 1 indicating complete inability to consume food or liquids orally.^10^ A computed tomography scan of the chest was performed before endoscopic intervention that did not reveal any mass lesion or evidence of recurrent malignancy. Endoscopy revealed complete obstruction at 16 cm from the incisors, just below the pyriform fossa. A Multidisciplinary Team meeting, involving thoracic surgeon and gastroenterologist, evaluated both surgical and endoscopic management options. The team opted for CARD, considering it the best approach to improve swallowing function with minimal invasiveness. The patient underwent CARD under general anesthesia. After removal of previously placed percutaneous endoscopic gastrostomy (PEG) tube, 2 ultrathin gastroscopes (Olympus GIF-XP190, outer diameter: 5.8 mm) were introduced: an antegrade scope through the mouth to the obstruction and a retrograde scope through the PEG tract to the lower end of the obstruction. Transillumination was performed by using the light from the retrograde endoscope to illuminate the tissues, helping to locate the position of the antegrade gastroscope, ensuring precise alignment of both scopes to avoid potential complications in this technically challenging location. A needle-knife was used through the retrograde scope to puncture the fibrotic tissue. A 0.035-inch guidewire (VisionGuide; Boston Scientific) was passed across the created tract and retrieved via the antegrade scope with grasping forceps, securing both ends. Sequential Savary-Gilliard dilators 5–11 mm (Cook Medical) were advanced, establishing luminal patency (Figures 1 and 2). Postdilatation, an adult gastroscope (GIF-H190) was passed without difficulty. The stricture was seen extending till 19 cm from the incisors, with a total length of 3 cm. The PEG tube was replaced through the same previous tract. The total fluoroscopy time was 4 minutes. No immediate complications occurred. The patient underwent 2 additional dilatations after the initial CARD procedure, during which restenosis was not observed, and a luminal diameter of 15 mm was achieved. The PEG was removed after the second session of dilatation. The patient's nutritional status was monitored through albumin levels and body weight, both of which improved after the procedure. At the 1-year follow-up, he is tolerating a soft diet (FOIS score 6), although his ability to consume a regular diet remains limited because of postsurgical mastication difficulties.

### Case 2

**Figure 3.**
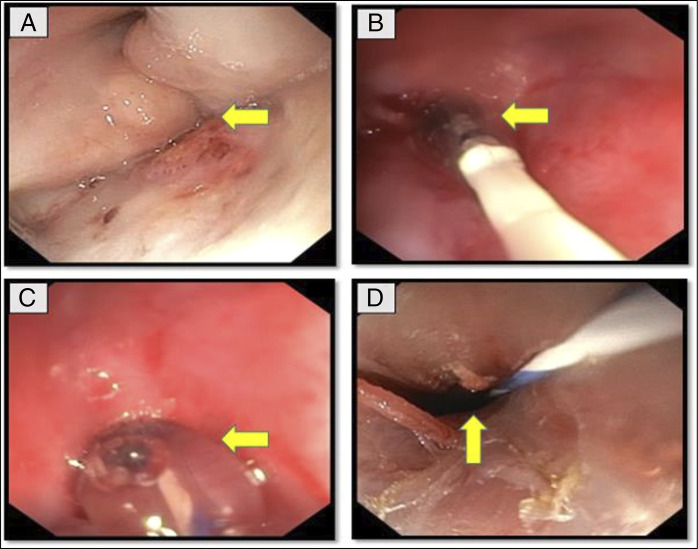
Endoscopic views of antegrade scope in CASE 2 with arrow head showing: (A) complete esophageal obstruction; (B) dilatation through hurricane balloon dilator; (C) CRE balloon dilatation; and (D) recanalization of esophageal lumen after balloon dilatation.

**Figure 4.**
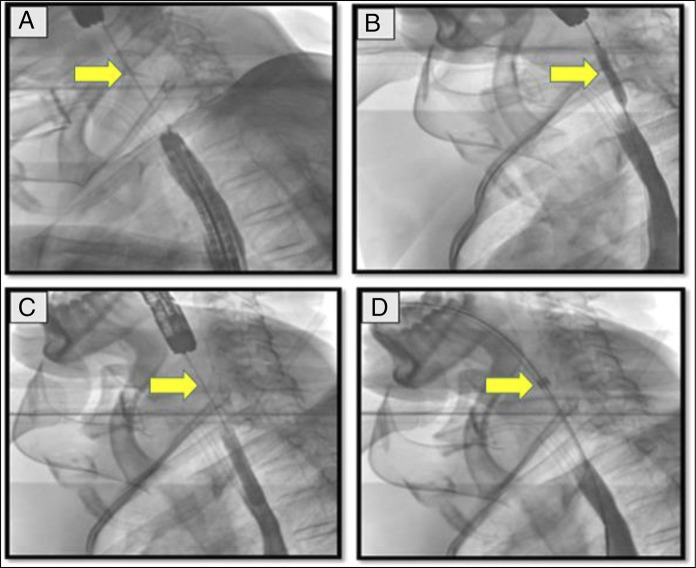
Fluoroscopic images of CASE 2 with arrow head showing: (A) guidewire between antegrade and retrograde scope; (B) balloon dilatation; (C) guidewire in place for dilatation; and (D) dilatation through Savary-Gilliard dilator.

A 15-year-old boy with hypopharyngeal squamous cell carcinoma, initially treated with 7 cycles of chemotherapy and 35 sessions of radiotherapy, followed by additional chemoradiotherapy for residual disease, presented with an 8-month history of complete dysphagia (FOIS score 1). A computed tomography scan of the chest was performed as part of the evaluation that did not demonstrate any mass lesion or alternative cause of obstruction. Endoscopy revealed complete obstruction at 15 cm from the incisors, near the cricopharyngeus. A Multidisciplinary Team meeting, including thoracic surgery and gastroenterology specialists, was convened to assess the management options. After evaluating the location of stricture, severity of narrowing, and the patient's clinical status, the team opted for endoscopic dilatation over surgery because of its less invasive nature and the potential to improve swallowing function with fewer risks. CARD was performed after the removal of the PEG tube. An ultrathin endoscope (Olympus GIF-XP190) was introduced retrograde via the gastrostomy, whereas an antegrade therapeutic scope (GIF-2TH180) was inserted orally. Under transillumination guidance, a 19-gauge fine needle aspiration needle (Boston Scientific) was used to puncture the stricture, followed by introduction of a 6 Fr cystotome (Cook Medical) over a 0.035-inch guidewire (VisionGuide; Boston Scientific). Sequential dilation was performed using a 4-mm hurricane balloon (Boston Scientific). After initial balloon dilation, resistance was encountered, and the stricture did not achieve adequate patency. Given the fibrotic nature of the stricture, a 10 Fr Soehendra dilator (Cook Medical) was used because of its ability to handle tight, fibrotic obstructions. Tactile resistance confirmed the need for a more aggressive approach. After this, an 8-mm controlled radial expansion (CRE) balloon (12–18 mm Hg pressure) was used to further expand the stricture, as fluoroscopic guidance and resistance indicated the need for additional dilation. Each step was guided by fluoroscopy and tactile feedback to ensure effective and safe dilation (Figures 3 and 4). The stricture was seen extending till 20 cm from the incisors, with a total length of 5 cm. A 14 Fr nasogastric (NG) tube was placed across the lumen to maintain luminal patency, while PEG feeding was continued. Two weeks later, repeat dilation was performed and the NG tube was removed. Although oral intake improved, PEG tube dependence persisted. After 1 dilatation post-CARD, repeat endoscopy during the second session again revealed CEO. CARD was repeated by retrograde guidewire passage through the stricture, which was then grasped with an antegrade ultrathin scope (Olympus GIF-XP190). A 5 Fr catheter was advanced, and dilation performed sequentially with an 8-mm CRE balloon (12–18 mm Hg pressure), followed by 5-mm and 7-mm Savary-Gilliard dilators (Cook Medical). Mild bleeding occurred but resolved spontaneously. An NG tube was kept in place, meanwhile feeding was continued through the PEG tube. The fluoroscopy time was 5 minutes. After the first 3 sessions of dilatation, during which restenosis was observed but CEO did not recur, the NG tube was removed, and patient was allowed to resume oral intake. The patient underwent a total of 11 sessions of dilatation after the second CARD procedure, during which a luminal diameter of 15 mm was gradually achieved. The sequential dilatation sessions were initially performed every 2 weeks, and later the interval was extended to 3 to 4 weeks, depending on the patient's symptoms. The patient's nutritional status was monitored through albumin levels and body weight, which showed some improvement after the procedure. Oral intake improved to liquids (FOIS 3), but PEG tube dependence persisted at the last follow-up.

## DISCUSSION

CEO is a challenging late complication of chemoradiotherapy for head and neck cancers.^11^ Both of our patients presented with radiation-induced CEO, 1 in adulthood and 1 in adolescence. Both suffered from absolute dysphagia with dependence on PEG feeding.

Traditionally, CEO was managed surgically or through complex multidisciplinary interventions, but CARD has emerged as a minimally invasive and safer alternative.^12,13^ Dellon et al reported successful outcomes in radiation-induced strictures treated with CARD, with improved swallowing and avoidance of esophagectomy.^14^ A systematic review by Jayaraj et al of 19 studies (229 procedures) showed 88% technical and 58% clinical success.^8^

Our first case demonstrates successful recanalization of a proximal stricture using ultrathin scopes, trans-illumination, needle-knife puncture, and graded Savary dilators, achieving PEG independence without complications. This outcome aligns with literature where up to 43% of patients resume oral intake after CARD.^8^

The second case highlights challenges in pediatric patients with complex strictures. Despite a narrow, fibrotic esophagus, tailored CARD with balloon, Soehendra, and CRE dilators achieved modest gains. During the procedure, mild self-limited bleeding occurred, which resolved spontaneously without the need for any therapeutic intervention. According to the complication classification by Jayaraj et al, this was categorized as a minor complication.^8^ This is consistent with the typical incidence of minor bleeding (6%–10%) reported in similar endoscopic procedures.^15^ Recurrence necessitated repeat CARD and serial dilatations, reflecting the high risk in radiation-induced CEO from fibrosis and poor tissue elasticity.^16^ Recovery was limited (FOIS 1 to 3, liquids only) with persistent PEG dependence, underscoring the challenges of proximal, fibrotic strictures near the hypopharynx. This case highlights the need for long-term multidisciplinary care and counseling.

From a quality-of-life perspective, studies have shown that even partial restoration of swallowing function can significantly improve social, nutritional, and emotional well-being, highlighting the importance of interventions such as CARD in enhancing patient outcomes.^17^ Dysphagia is known to negatively affect nutrition, social interactions, and emotional well-being.^18^ Our cases illustrate that CARD can provide meaningful functional improvement even when PEG dependence persists. However, CARD carries risks, including perforation (up to 8%) and frequent recurrence, with repeated dilatations reflecting the chronic nature of postradiation fibrosis and the need for long-term follow-up.^8^

CARD is an effective, minimally invasive technique for managing radiation-induced CEO, especially in difficult locations near the upper esophageal sphincter. It avoids the morbidity of surgical reconstruction, restores swallowing, reduces PEG dependence, and improves quality of life. However, recurrence and the need for repeat interventions remain important limitations, requiring careful patient selection, counseling, and long-term follow-up.

## DISCLOSURES

Author contributions: All authors made significant contributions to the work. M. Siyal wrote the case report and reviewed the literature. AR Siddiqui and S. Kadir contributed to the review of the article. SN Niaz performed critical review, editing and provided final approval of the manuscript. All authors have reviewed and approved the final version and agree to be accountable for all aspects of the work. SK Niaz accepts full responsibility for the integrity and accuracy of the work as a whole. SK Niaz is the article guarantor.

Financial disclosure: None to report.

Previous presentation: This case report has not been presented previously at any professional or academic meeting.

Informed consent was obtained for this case report.

## ABBREVIATIONS:

CARD: combined antegrade and retrograde endoscopic dilatation
CEO: complete esophageal obstruction
PEG: percutaneous endoscopic gastrostomy
